# How socioeconomic status influences self-care for Black/African American women: A differential item analysis

**DOI:** 10.1016/j.pmedr.2020.101155

**Published:** 2020-07-02

**Authors:** Paris B. Adkins-Jackson

**Affiliations:** Center for Health Policy Research, Fielding School of Public Health, University of California, Los Angeles, 10960 Wilshire Boulevard, Suite 1550, Los Angeles, CA 90024, United States

**Keywords:** Self-care, Socioeconomic status, Black women, Differential item functioning

## Abstract

It is well documented that Black/African American (Black) women's health is impacted by socioeconomic status (SES). Self-care can improve health but this is also contingent upon SES; though it is unclear which activities are impacted. This cross-sectional study performed differential item functioning with 47 self-care activities rated by 223 Black women. Participants were compared across activities by SES group—a composite score encompassing personal income, highest degree obtained, employment status, and number of dependents. Findings suggest SES impacts half of self-care activities with most being practiced significantly more by Black women with the highest SES as opposed to the lowest. The activities that were most influenced by SES included striving for balance, scheduling regular activities with their children, and finding ways to laugh. Such differences speak to the impact of SES on the overall health of Black women. Research on Black women's health would benefit from targeting the impact of SES on self-care in order to better increase the health of Black women.

## Highlights

• Income, highest degree obtained, employment status, and number of dependents make SES.
• SES impacts 49% of self-care activities that Black women engage.
• The disparity in self-care exists between the lowest and highest SES.
• SES impacts balance, time, and laughing.

## 1 Introduction

Literature has consistently shown that Black/African American (Black) women disproportionately experience poor health relative to white women (National Institutes of Health, Office of Research on Women’s Health, 2014). Such experiences of poor health occur due to socioeconomic influences such as income, education, and employment (Krishnan et al., 2010). Additional literature suggests enduring socioeconomic challenges while caring for others also impact the health of Black women, which is particularly salient for Black women who are expected by their community to reflect strength, and to their suppress emotions and feelings of vulnerability (Suplee et al., 2015, Woods-Giscombe, 2010).

Fortunately, the literature also suggests self-care can improve health (Adkins-Jackson et al., 2019). Self-care is the application of a variety of activities, mind and body related, that are intentionally employed to restore health or prevent disease (Denyes et al., 2001, Chang et al., 2005, Li et al., 2010, Kinser et al., 2016, Shanklin-Flowers, 2012). This literature suggests personal income and education are positively associated with self-care, and that there is a positive linear relationship between number of dependents, a proxy for resilience, and self-care (Adkins-Jackson et al., 2019). The latter finding implies that as the number of individuals a Black woman cares for increases, the greater her self-care (Adkins-Jackson et al., 2019). The inverse of this relationship was reported by Suplee et al. (2015) as they observed that women of color practice self-care less due to caring for others.

Despite the aforementioned role of income, education, and number of dependents in the application of overall self-care, minimal literature has examined which self-care activities are impacted by a combination of socioeconomic factors (Suplee et al., 2015). Given limited research, this study sought to examine if increases in socioeconomic status (SES) influence the utilization of specific self-care practices. Using a combined inventory of self-care activities (Saakvitne and Pearlman, 1996, Butler, 2010), this study asked which self-care practices differ based on SES. It was hypothesized that a majority of items would differ based on existing literature that suggests there are positive relationships between self-care and income, education, and number of dependents (Suplee et al., 2015, Adkins-Jackson et al., 2019).

## 2 Methods

### 2.1 Study design

A link to an online questionnaire with a prompt requesting the participation of only Black women was emailed to offices of institutional research at universities (e.g., minority-serving institutions) across the United States, as well as to human resources offices at national organizations with large Black constituents and/or employees (e.g., sororities). Additionally, a link to the survey was posted on social media (e.g., Facebook). Snowball sampling was encouraged in the prompt and at the end of the questionnaire. The questionnaire included screening questions to ensure the criteria of being Black, female, and 18 or older were met, in addition to a consent form that required participants to agree to the participate before moving forward with study questions. Completion of the full questionnaire was estimated at 20–30 min and it was available for five months. The Institutional Review Board at a university approved this study in fall 2016.

There were 223 adult Black women who participated in this study (see Table 1). Participants had an average age of 35, were heterosexual (86.5%), single (62.6%), and were non-immigrants (92.8%). Most participants had non-immigrant parents or grandparents (84.8%) and did not have dependents (62.6%). Participants were employed full-time (52.5%), with personal income between $0–25,000 (44.4%) and the most frequent highest degrees obtained were high school diplomas (32.7%) and graduate master’s degrees (26.9%). Geographic location of participants was not collected.

**Table 1:** Demographic profile of survey participants.

Category	Mean *(SD)*	Frequency	Percentage %
Age	34.86 *(14.86)*	222	99.6
Category		223	100.0
Immigration			
Non-immigrant		207	92.8
Parental Immigration			
Non-immigrant		189	84.8
Sexual orientation			
Heterosexual		193	86.5
Bisexual, Homosexual, or other		30	13.4
Marital status			
Single		139	62.6
Married/ domestic partnership		50	22.5
Divorced		30	13.5
Dependents			
0		139	62.6
1		42	18.9
2 or more		41	18.5
Employment status			
Full time		117	52.5
Part-time		51	22.9
Unemployed		40	17.9
Highest degree			
Diploma/GED		73	32.7
Associates		24	10.8
Bachelors		40	17.9
Masters		60	26.9
Doctorate		26	11.7
Personal income			
$0–25,000		99	44.4
$26–50,000		43	19.3
$51–75,000		38	17.0
$76–100,000		22	9.9
>$101,000		21	9.4

### 2.2 Measures

#### 2.2.1 Socioeconomic variables

Participants completed an online battery of nine behavioral assessments with a demographic section that collected four measures that constituted the measure of SES: personal income, highest degree obtained, employment status, and number of dependents. Response categories for these variables were as follows: Personal income – $0–25,000, $26–50,000, $51–75,000, $76–100,000, or $101,000 and above; Highest degree obtained – High school diploma or GED, Bachelor’s degree, Master’s degree, or Doctoral degree; Employment status – Not employed, part-time employed, full-time employed; and, Number of dependents – 0, 1, and 2 or more.

#### 2.2.2 Combined inventory of self-care activities

Saakvitne, Pearlman, and Staff of the Traumatic Stress Institute/Center for Adult and Adolescent Psychotherapy (Saakvitne and Pearlman, 1996) developed an assessment with 65 self-care activities for clinicians who experience vicarious trauma from working with clients who experience traumatic events. Items include: “eat healthy,” “get enough sleep,” “make time for reflection,” “say ‘no’ to extra responsibilities,” “spend time with others whose company you enjoy,” “allow yourself to cry,” “find a spiritual connection,” and “set limits with your clients and colleagues.” The assessment was adapted by Butler (2010) who increased the assessment to 72 items.

Response categories for the combined inventory of self-care activities were: *0 = “not applicable/it didn’t occur to me/never,” 1 = “rarely,” 2 = “occasionally,” and 3 = “frequently.”* A previous study with a parallel sample of 12 Black women subject-matter experts determined 47 items on the combined assessment were content valid with Black women (Adkins-Jackson, 2018). The experts practice self-care and offer self-care services (e.g., therapy, reiki, yoga, blog posts, wellness programs, etc.) to other Black women. These experts reviewed items on the combined inventory of self-care activities and selected items they considered to be “relevant” to Black women’s self-care. Items were determined to be content-valid if a majority (58%) of experts deemed them relevant. Subsequently, only these items were used in this study. The assessment obtained strong reliability (α = 0.911). The mean per item and frequencies per response category are reported in Table 2.

**Table 2:** Descriptive statistics and reliability for self-care assessment items (K = 47).

Label	Mean (*SD*)	Alpha	NA/Didn’t occur/Never		Rarely		Occasionally		Frequently	
			#	%	#	%	#	%	#	%
Eat regularly	3.96 (*0.842*)	0.908	9	4.0	54	24.2	97	43.5	63	28.3
Eat healthy	4.54 (*0.696*)	0.908	3	1.3	14	6.3	65	29.1	141	63.2
Exercise	4.43 (*0.719*)	0.909	3	1.3	15	6.7	86	38.6	119	53.4
Get regular med. care prevention	3.87 (*0.946*)	0.907	15	6.7	61	27.4	81	36.3	66	29.6
Get medical care when needed	3.90 (*0.864*)	0.908	13	5.7	46	20.6	111	49.8	53	23.8
Take time off when needed	3.45 (*0.878*)	0.908	23	10.3	86	38.6	96	43.0	18	8.1
Dance et al.	3.82 (*0.856*)	0.909	9	4.0	72	32.3	90	40.4	52	23.3
Get enough sleep	4.30 (*0.724*)	0.909	3	1.3	26	11.7	96	43.0	98	43.9
Wear clothes you like	4.09 (*0.984*)	0.908	13	5.8	47	21.1	65	29.1	98	43.9
Take vacations	4.54 (*0.695*)	0.909	4	1.7	11	4.9	67	30.0	141	63.2
Take day trips or mini-vacations	4.17 (*0.909*)	0.908	9	4.0	34	15.2	85	38.1	95	42.6
Make time for self-reflection	4.38 (*0.850*)	0.909	5	2.1	23	10.3	73	32.7	122	54.7
Write in a journal	4.56 (*0.668*)	0.908	1	0.4	16	7.2	63	28.3	143	64.1
Do something you are not expert	4.18 (*0.851*)	0.907	6	2.7	40	17.9	83	37.2	94	42.2
Decrease stress in your life	3.95 (*1.09*)	0.909	23	10.2	46	20.6	66	29.6	88	39.5
Notice your inner experience	4.55 (*0.714*)	0.909	3	1.3	14	6.3	62	27.8	144	64.6
Engage your intelligence	4.03 (*1.10*)	0.910	17	7.6	32	14.3	87	39.0	87	39.0
Practice receiving from others	3.98 (*0.900*)	0.908	11	4.8	43	19.3	103	46.2	66	29.6
Be curious	2.74 (*1.93*)	0.916	83	37.2	41	18.4	47	21.1	52	23.3
Find a spiritual connection	3.89 (*0.879*)	0.909	9	4.0	65	29.1	88	39.5	61	27.4
Stay in contact w/ impt. people	4.28 (*1.03*)	0.910	8	3.5	35	15.7	55	24.7	125	56.1
Give yourself affirmations	4.61 (*0.694*)	0.910	2	0.8	11	4.9	55	24.7	155	69.5
Love yourself	3.77 (*1.01*)	0.909	17	7.6	68	30.5	80	35.9	58	26.0
Re-read favorite books/movies	3.36 (*0.919*)	0.908	33	14.8	89	39.9	82	36.8	19	8.5
Identify comforting activities et al.	3.40 (*1.02*)	0.907	36	16.1	85	38.1	69	30.9	33	14.8
Allow yourself to cry	3.17 (*1.16*)	0.911	80	35.8	55	24.7	50	22.4	38	17.0
Find things that make you laugh	3.74 (*0.974*)	0.909	16	7.2	65	29.1	93	41.7	49	22.2
Make time for reflection	4.34 (*0.800*)	0.908	4	1.7	21	9.4	89	39.9	109	48.9
Spend time with nature	4.38 (*0.807*)	0.907	4	1.7	28	12.6	68	30.5	123	55.2
Spend time with others you enjoy	3.74 (*0.987*)	0.909	25	11.2	63	28.3	77	34.5	58	26.0
Be open to inspiration	4.11 (*0.868*)	0.907	7	2.1	40	17.9	94	42.2	82	36.8
Cherish your optimism and hope	3.88 (*0.932*)	0.910	13	4.7	56	25.1	93	41.7	61	27.4
Be aware of nonmaterial aspects	4.37 (*0.822*)	0.908	9	4.0	19	8.5	75	33.6	120	53.8
Take a break during the workday	4.46 (*0.873*)	0.910	6	2.6	16	7.2	64	28.7	137	61.4
Make quiet time to complete tasks	4.04 (*1.25*)	0.909	21	8.4	31	13.9	66	29.6	105	47.1
Identify projects that are exciting	3.91 (*1.00*)	0.908	19	8.5	53	23.8	77	34.5	74	33.2
Set limits with clients/colleagues	4.50 (*0.690*)	0.909	1	0.4	19	8.5	69	30.9	134	60.1
Balance your caseload	3.86 (*0.909*)	0.907	10	4.4	61	27.4	97	43.5	55	24.7
Arrange your work space	3.79 (*0.928*)	0.907	19	8.5	54	24.2	101	45.3	49	22.2
Schedule regular dates w/ partner	3.61 (*0.984*)	0.908	22	9.8	67	30.0	98	43.9	36	16.1
Schedule reg. activities w/ children	3.61 (*0.928*)	0.908	22	9.8	81	36.3	79	35.4	41	18.4
Make time to see friends	4.30 (*0.830*)	0.908	5	2.1	28	12.6	82	36.8	108	48.4
Call, check on, or see my relatives	3.74 (*1.30*)	0.909	27	12.1	43	19.3	84	37.7	69	30.9
Allow others to do things for me	3.71 (*1.05*)	0.909	19	8.5	61	27.4	94	42.2	49	22.0
Ask for help when I need it	1.96 (*2.21*)	0.919	129	57.8	7	3.1	36	16.1	51	22.9
Share a fear/hope/secret	4.16 (*0.839*)	0.909	8	3.5	26	11.7	107	48.0	82	36.8
Strive for balance et al.	4.33 (*0.825*)	0.908	5	2.1	26	11.7	80	35.9	112	50.2

### 2.3 Statistical analyses

#### 2.3.1 SES variable

The SES variables—employment, highest degree obtained, number of dependents, and personal income—were put into a principal axis exploratory factor analysis (EFA) with a Promax rotation using SPSS Version 22 (IBM Corp, 2013) and transformed into factor scores. The factors scores produced a continuous SES variable that was partitioned into quartile groups: low SES, low-mid SES, mid-high SES, and high SES (Krishnan et al., 2010) (see Table 3).

**Table 3:** Factor loadings and communalities for SES variables and frequency of SES groups.

Items	Factor 1	Communalities
Personal income	0.873	0.762
Highest degree	0.767	0.588
Employment	0.723	0.523
Number of dependents	0.430	0.185

Group	Group Label	Factor Scores	Frequency	Percent
1	Low SES	−2, −0.8262	56	25.1
2	Low-mid SES	−0.8262, −0.0790	55	24.7
3	Mid-high SES	−0.0790, 0.7600	55	24.7
4	High SES	0.7600, 2	56	25.1

#### 2.3.2 Self-care by SES

Given both SES and self-care employed Likert scales with ordinal polytomous items, differential item functioning (DIF) was used to perform these analyses. DIF is a psychometric item analysis approach that compares groups per item for ordinal multinomial dependent and independent variables (Holland and Thayer, 1986). Item response theory via Rasch measurement was used to reduce error by standardizing and transforming response categories from an ordinal scale (e.g., 1 = “rarely,” 2 = “occasionally,” etc.) to a continuous scale between −2 to 2.

DIF uses the mean self-care response per item for a SES group (e.g., for given item “eat healthy” mean for the low SES group is −2 and the mean for high SES group is 2) as fixed numbers to calculate Mantel-Haenszal chi-square values (Zwick and Thayer, 1996). Each item on the combined inventory of assessments were compared between two SES groups at a time. Consequently, all items were compared between low and low-mid SES groups, then all items were compared between low and mid-high SES groups, and the same for all possible comparisons: low and high SES groups, low-mid and mid-high SES groups, low-mid and high SES groups, and mid-high and high SES groups. A statistically significant (*p* < .05) chi-square test statistic greater than 1 indicates there are differences between groups. Given multiple comparisons, significant p-values were adjusted using the Bonferroni-Holm method. DIF analyses were performed using Winsteps software (Linacre, 2017) and Bonferroni-Holm using Microsoft Excel.

## 3 Results

### 3.1 SES

The EFA produced slight concerns with the factorability of the SES measure (Kaiser-Meyer-Olkin measure of sampling of adequacy = 0.756), but there were sufficient correlations between the four variables [Barlett’s test*, χ*^2^ (6) = 292.03, *p* = .000] (Meyers et al., 2013). All four variables loaded onto one factor with no other factors extracted (eigenvalue = 2.47, total variance explained 61.80%) (see Table 3). *Personal income* loaded the highest (0.873) and *number of dependents* (0.430) the lowest. Based on these factor loadings, a SES factor score was computed for each participant.

### 3.2 Self-care activities

Forty-nine percent of the items (*K* = 23) had statistically significant differences between SES groups (see Table 4). These significant findings were confirmed after p-value adjustment. There were only positive chi-square values obtained in these analyses.

**Table 4:** Chi-square values above 1 for significantly different SES groups per item.

Items	SES Groups					
	Low & Low-Mid	Low & Mid-High	Low & High	Low-Mid & Mid-High	Low-Mid & High	Mid- High & High
Eat regularly						
Eat healthy		3.90*			6.42*	
Exercise		5.07*				7.40**
Get regular med. care prevention		5.10*				
Get medical care when needed		*3.67*	4.39*			
Take time off when needed						
Dance et al.						
Get enough sleep	4.95*	*3.49*				
Wear clothes you like						
Take vacations		4.17*				
Take day trips or mini-vacations		*3.81*	7.59**		6.57*	
Make time for self-reflection						
Write in a journal					4.45*	
Do something you are not expert						
Decrease stress in your life			4.86*			
Notice your inner experience						
Engage your intelligence						
Practice receiving from others						
Be curious	*3.42*			5.94*		
Find a spiritual connection		5.73*		5.26*		
Stay in contact w/ impt. people			*3.73*			
Give yourself affirmations				*3.34*		
Love yourself						
Re-read favorite books/movies						
Identify comforting activities et al.		*3.45*	6.55*			
Allow yourself to cry			*3.62*			
Find things that make you laugh		8.28**	10.40**		4.47*	
Make time for reflection						
Spend time with nature						
Spend time with others you enjoy		*3.58*		6.85**		
Be open to inspiration						
Cherish your optimism and hope						
Be aware of nonmaterial aspects		4.30*				
Take a break during the workday			7.64**			
Make quiet time to complete tasks						
Identify projects that are exciting						
Set limits with clients/colleagues						*3.44*
Balance your caseload						5.66*
Arrange your work space						
Schedule regular dates w/ partner			7.73**			
Schedule reg. activities w/ children	8.73**	5.60*	8.48**			
Make time to see friends		4.23*	9.88**			
Call, check on, or see my relatives						
Allow others to do things for me						
Ask for help when I need it		4.91*				
Share a fear/hope/secret					8.14**	
Strive for balance et al.		6.93**	6.96**	*3.64*	4.53*	4.09*

*# of Differences (p < .05)*	*2*	*11*	*10*	*3*	*6*	*3*
*# of Marginal Differences (p* > .05 < 0.10*)*	*1*	*5*	*2*	*2*	*0*	*1*

Italicized *p* > .05 < 0.10; *p < .05; **p < .01.

#### 3.2.1 Low and Low-mid SES

**Fig. 1:**
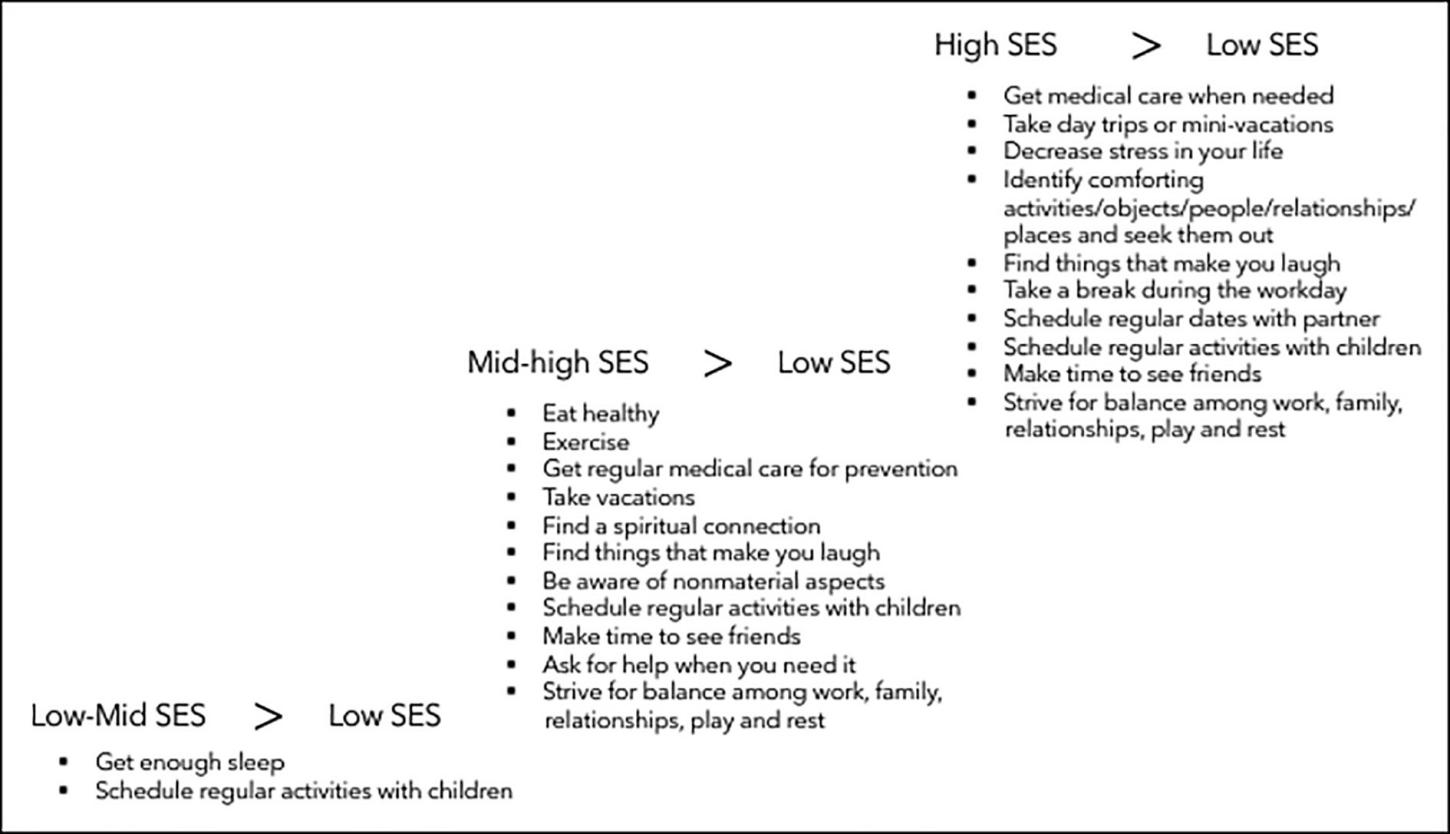
Comparison of self-care activities between Black women with low SES and other SES groups.

Significant differences existed on the following items: *Get enough sleep* and *Schedule regular activities with children* (Fig. 1).

#### 3.2.2 Low and mid-high SES

Eat healthy, Exercise, Get regular medical care for prevention, Take vacations, Find a spiritual connection, Find things that make you laugh, Be aware of nonmaterial aspects, Schedule regular activities with children, Make time to see friends, Ask for help when I need it, and Strive for balance among work, family, relationships, play and rest.

#### 3.2.3 Low and high SES

Get medical care when needed, Take day trips or mini-vacations, Decrease stress in your life, Identify comforting activities/objects/people/relationships/places and seek them out, Find things that make you laugh, Take a break during the workday, Schedule regular dates with partner, Schedule regular activities with children, Make time to see friends, and Strive for balance among work, family, relationships, play and rest.

#### 3.2.4 Low-mid and Mid-high SES

**Fig. 2:**
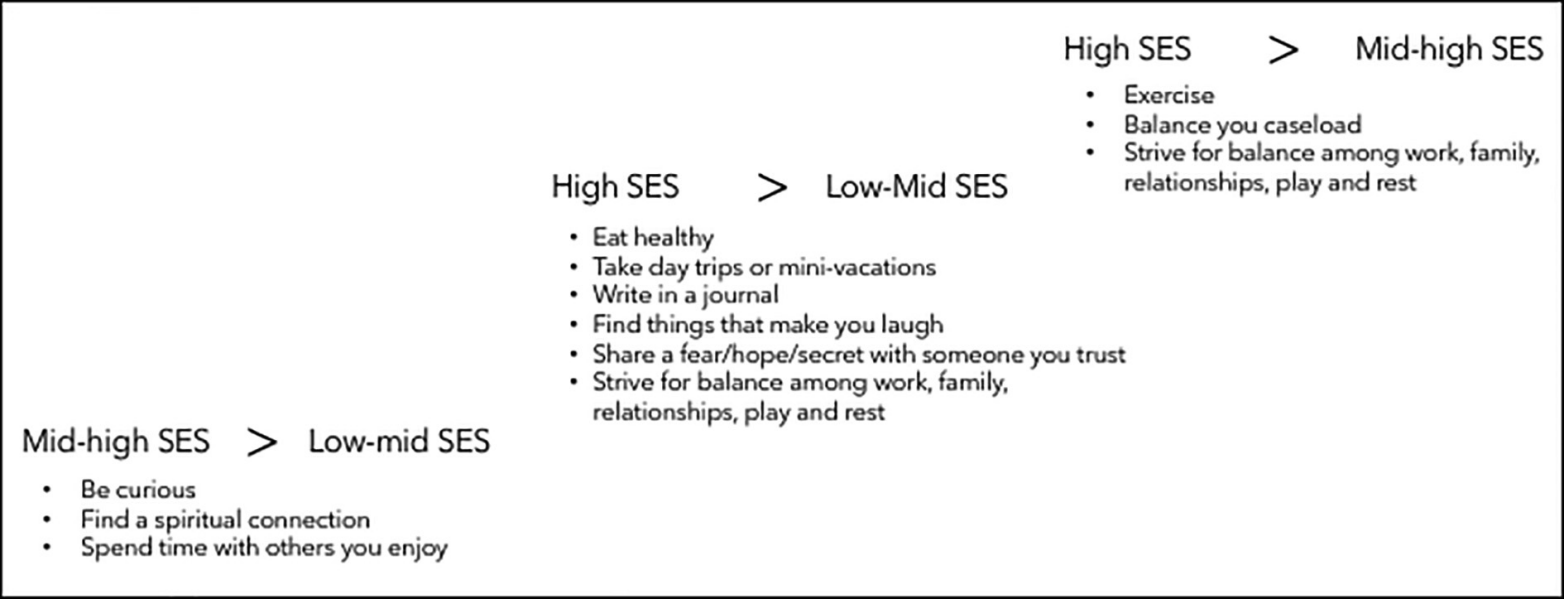
Comparison of self-care activities between SES groups.

Be curious, Find a spiritual connection, and Spend time with others you enjoy (Fig. 2).

#### 3.2.5 Low-mid and high SES

Eat healthy, Take day trips or mini-vacations, Write in a journal, Find things that make you laugh, Share a fear/hope/secret with someone I trust, and Strive for balance among work, family, relationships, play and rest.

#### 3.2.6 Mid-high and high SES

Exercise, Balance your caseload, and Strive for balance among work, family, relationships, play and rest.

#### 3.2.7 Items with the most frequent SES differences

*Strive for balance among work, family, relationships, play* and rest, *Scheduling regular activities with my children*, and *Finding things that make you laugh* frequently had significant differences between SES groups. *Strive for balance among work, family, relationships, play and rest* differed by each SES group except for between the lowest SES groups [low and low-mid SES (p = .815)] and the middle SES groups [low-mid to mid-high SES (p = .056)].

*Scheduling regular activities with my children* had three significant differences including between the lowest SES group and the highest SES groups (low and mid-high SES, low and high SES). There was also a significant difference between the lowest SES groups (low and low-mid SES) on this item. Similarly, *Finding things that make you laugh* had three significant differences including those between the lowest SES groups and the highest SES groups (low and mid-high SES, low and high SES, low-mid and high SES).

## 4 Discussion

This research examined the impact of SES on self-care by using DIF to compare SES groups by self-care activity from a combined inventory of self-care items (Saakvitne and Pearlman, 1996, Butler, 2010). Statistically significant positive chi-square values above 1 indicated the greater the SES, the more the self-care activity was practiced. The study’s hypothesis was supported that increases in SES would result in greater self-care for Black women. This hypothesis was evidenced by 49% of self-care activities significantly differing by SES. Consequently, these findings substantiate the literature that self-care is greatly impacted by SES for Black women (Suplee et al., 2015, Adkins-Jackson et al., 2019).

Most statistically significant differences existed between the two lowest SES groups and the highest SES group. This suggests there are disparities in SES that dictate the amount of self-care Black women get to practice. There are expected differences for activities that require money (e.g., *Take day trips or mini-vacations*, *Take vacations*) and/or time (e.g., *Write in a journal,* maintain personal relationships with friends, spouses, children, and other important people). Given existing physical and mental health disparities, there are expected differences in behaviors related to health and balance (e.g., *Eat healthy*, *Get medical care when needed*, *Decrease stress in your life*) (National Institutes of Health, Office of Research on Women’s Health., 2014, Alliance, 2015). Arguably, there are expected disparities in the activity with the most frequent significant differences, *Strive for balance among work, family, relationships, play and rest*, as the higher the SES, the more a woman strives for balance. While greater SES provides more access to resources to assist with managing one’s life, it does not necessarily reduce the effort exerted to balance them (Mullings, 2005). Subsequently, although these self-care differences influence the health and balance of Black women, there is existing literature that suggest such differences occur.

Conversely, there are differences between low and high SES groups that are not previously discussed in the literature. *Find things that make you laugh* was the second item with the most frequent significant differences between SES groups, with only no difference between mid-high and high SES. This suggests finding a way to laugh is limitedly accessible to Black women in SES groups below mid-high. Similarly, *Identify comforting activities et al. and seek them out, Allow yourself to cry*, and *Share a fear/hope or secret with someone I trust* differed significantly between the lowest SES groups and the highest SES group. These actions were not discussed in previous literature to determine a rationale for why they are contingent upon socioeconomic factors. Subsequently, these differences need to be explored further. It is possible there were other elements of these self-care items, such as wording, that influenced how participants responded.

Additionally, much of literature on the Superwoman Schema, and other related experiences, describe vulnerability as difficult for Black women for a myriad of reasons often related to SES (Woods-Giscombe, 2010, Shanklin-Flowers, 2012). Subsequently, allowing one’s self to cry and sharing a secret may not be activities culturally-appropriate for this population’s self-care. For *Allow yourself to cry*, 36% of participants never practiced the activity, considered it not applicable, or never considered it at all. It is possible through the infrequency of this activity that it was considered irrelevant to self-care for participants. Contrarily, both *Be curious* and *Ask for help when I need it* also had high frequencies of not applicable/it didn’t occur to me/never practiced, but these activities did not yield the amount of significant differences between SES groups that the former did.

There were a number of self-care activities that did not differ between SES groups. Thus, SES does not interfere with Black women practicing activities from the psychological, emotional, and professional self-care subscales of the combined inventory of self-care activities (with a few exceptions). A list of these activities were provided in Table 4.

Interestingly, number of dependents predicted self-care in a previous study (Adkins-Jackson et al., 2019), but had a small impact on this measure of SES. This is evident in the low factor loading of this variable as it shared enough variance with other variables to load onto the factor, but as communalities suggest, this item is poorly accounted for by the scale. It could also be assumed that this variable should negatively load onto the measure of SES as more dependents are expected to provide a financial tax. However, in this sample with largely Black women with no dependents, an inverse relationship was not derived. Future research may consider how to incorporate this variable into a measure of SES.

### 4.1 Limitations

Limitations to this study include the cross-sectional nature of the research. This study was observational and limited causal inferences. Additionally, the study questionnaire did not include a way to confirm the national representativeness of the sample. Lastly, these assessments were created for social workers and were not specified for use with Black women. Subsequently, the self-care activities provided may not have been culturally-responsive for Black women.

### 4.2 Conclusion

Many of the self-care activities practiced by Black women were influenced by SES with most of the differences existing between the lowest and highest SES groups. These differences reveal disparities in self-care, which may impact whether Black women seek to practice self-care. With a majority of Black women living below the poverty line (Bureau, 2015), and numerous studies showing the negative influence of SES on health; these findings suggests a remedy for poor health (i.e., self-care) is also negatively influenced by SES. DIF was used to illuminate these relationships by reducing the error that could occur from using statistical methods inappropriate for multiple polytomous variables. Subsequently, the intent of using these methods were to make clear the challenges to health Black women face and to encourage programmatic and policy changes to reduce the role of SES on health and self-care.
